# The incidence of early recurrent venous thromboembolism: a systematic review and meta-analysis

**DOI:** 10.1016/j.rpth.2025.103317

**Published:** 2025-12-29

**Authors:** Lisbeth Eischer, Paul A. Kyrle, Alexandra Kaider, Anton Schmidt, Brigitte Wildner, Anja Boc, Charlotte Bradbury, Anetta Undas, Francis Couturaud, Matteo Nicola Dario Di Minno, Geert-Jan Geersing, David Jimenez, Sameer Parpia, Gualtiero Palareti, Daniela Poli, Daniel P. Potaczek, Paolo Prandoni, Sam Schulman, Astrid van Hylckama Vlieg, Michal Zabczyk, Sabine Eichinger

**Affiliations:** 1 Department of Medicine I, Medical University of Vienna, Vienna, Austria; 2 Karl Landsteiner Institute of Thrombosis Research, Vienna, Austria; 3 Center for Medical Data Science, Institute of Clinical Biometrics, Medical University of Vienna, Vienna, Austria; 4 University Library, Medical University of Vienna, Vienna, Austria; 5 Institute of Anatomy, Faculty of Medicine, University of Ljubljana, Ljubljana, Slovenia; 6 Department of Vascular Diseases, University Medical Centre Ljubljana, Ljubljana, Slovenia; 7 Department of Hematology, Faculty of Health Sciences, University of Bristol, Bristol, United Kingdom; 8 Department of Thromboembolic Disorders, Institute of Cardiology, Jagiellonian University Medical College, Kraków, Poland; 9 Krakow Center for Medical Research and Technologies, The St. John Paul II Hospital, Kraków, Poland; 10 Chest Diseases, Centre Hospitalier Universitaire Brest, Institut national de la santé et de la recherche médicale (INSERM) U1304-GETBO, Brest, France; 11 Department of Clinical Medicine and Surgery, Federico II University, Naples, Italy; 12 Department of General Practice & Nursing Science, Julius Center for Health Sciences and Primary Care, University Medical Center Utrecht, Utrecht University, Utrecht, The Netherlands; 13 Department of Family Medicine, University of Washington, Seattle, Washington, USA; 14 Respiratory Department and Medicine Department, Ramon y Cajal Hospital (IRYCIS), and Alcala University Enfermedades Respiratorias (CIBERES), Madrid, Spain; 15 Department of Health Research Methods, Evidence, and Impact, McMaster University, Hamilton, Ontario, Canada; 16 Department of Oncology, McMaster University, Hamilton, Ontario, Canada; 17 Arianna Foundation on Anticoagulation, Bologna, Italy; 18 Thrombosis Centre, Azienda Ospedaliero-Universitaria Careggi, Firenze, Italy; 19 Center for Infection and Genomics of the Lung, Universities of Giessen and Marburg Lung Center, German Center for Lung Research, Giessen, Germany; 20 Translational Inflammation Research Division & Core Facility for Single Cell Multiomics, Medical Faculty, Philipps-University Marburg, Marburg, Germany; 21 Department of Medicine, McMaster University, Hamilton, Ontario, Canada; 22 Department of Clinical Epidemiology, Leiden University Medical Center, Leiden, The Netherlands

**Keywords:** anticoagulation, early recurrence, incidence, meta-analysis, venous thromboembolism

## Abstract

**Background:**

Patients with venous thromboembolism (VTE) receive anticoagulation for at least 3 months. To evaluate recurrence risk thereafter, some strategies include D-dimer testing after discontinuing anticoagulation, which raises concern about early recurrence.

**Objectives:**

To assess the incidence of recurrent VTE within 30 days after stopping anticoagulation.

**Methods:**

We conducted a systematic review of EMBASE, CENTRAL, and MEDLINE to identify controlled trials and cohort studies of adult noncancer patients with deep vein thrombosis of the leg and/or pulmonary embolism treated with anticoagulants for ≥3 months. The primary outcome was symptomatic VTE within 30 days. The risk of bias was assessed using a modified version of the Newcastle-Ottawa Scale. Pooled recurrence rates were calculated using fixed random-effects meta-analyses.

**Results:**

Of 42 studies, 24 (57%) provided data, encompassing 11,407 patients. Early recurrence occurred in 115 patients (1.01%), with a pooled incidence of 1.04% (95% CI, 0.8%-1.4%). Men had a risk similar to that of women (risk ratio, 1.2; 95% CI, 0.6-2.3; *P* = .7). Unprovoked VTE was associated with a 2.6-fold increase in risk (95% CI, 1.4-4.6; *P* < .001) compared with provoked VTE. Patients with deep vein thrombosis at presentation had a similar risk of recurrence compared with those with an incident pulmonary embolism (risk ratio, 0.6; 95% CI: 0.3-1.2; *P* = .1). Findings regarding age were inconsistent. None of the recurrences was fatal. The overall risk of bias was low.

**Conclusion:**

The incidence of early VTE recurrence after stopping anticoagulation is low. Temporarily discontinuing therapy to assess recurrence risk, therefore, appears safe and may aid in guiding treatment duration.

## Essentials

• Off-therapy D-dimer tests after VTE raise concern about early recurrence.
• We performed a systematic review and meta-analysis of studies of VTE patients anticoagulated for ≥3 months.
• Early recurrence was rare (1.04%). Stopping anticoagulation briefly appears safe.
• Off-therapy assessment may help guide treatment duration.

## 1 Introduction

Venous thromboembolism (VTE) is a common condition with a high risk of recurrence. Although anticoagulant therapy is effective in preventing recurrence, it also increases the risk of bleeding [1]. Therefore, long-term management of VTE requires a careful balance between the risk of recurrence and the potential for bleeding associated with anticoagulation.

In recent years, risk assessment models have been developed to improve the prediction of VTE recurrence and help optimize the duration of anticoagulant therapy [[2], [3], [4], [5], [6], [7], [8], [9], [10], [11]]. Several models, including VPM [3,4], DASH [5], L-TRRiP [6], Continu-8 [7], DAMOVES [8], and HERDOO2 [9], combine clinical risk factors with laboratory biomarkers of coagulation activation. To avoid the confounding effects of ongoing anticoagulation on these biomarkers, measurements are typically performed 3 to 4 weeks after therapy is discontinued. However, this approach introduces a critical period during which patients are potentially vulnerable to early recurrent thromboembolic events. For example, in one of our studies, 7 of 818 patients with a first unprovoked VTE experienced a recurrent event within 3 weeks of stopping anticoagulation [4].

This observation raises an important clinical question: is the temporary interruption of anticoagulation—required for the use of certain risk assessment models—justifiable, especially for patients at a potentially high risk of recurrence?

The aim of this systematic review was to evaluate the incidence of recurrent deep vein thrombosis (DVT) or pulmonary embolism (PE) within the first 30 days following the cessation of anticoagulant therapy, ie, the rate of early recurrence.

## 2 Methods

### 2.1 Protocol and registration

This systematic review was conducted in accordance with the Preferred Reporting Items for Systematic Reviews and Meta-Analyses guidelines [12,13]. The protocol was registered on the PROSPERO platform (registration number CRD42023373784) on March 19, 2023.

### 2.2 Eligibility criteria

We included full-text articles of controlled trials and prospective observational studies published between 1995 and 2022, with titles and abstracts available in English. Eligible studies had to report on the recurrence risk in adults with an objectively confirmed symptomatic episode of DVT of the lower limb and/or PE following discontinuation of at least 3 months of anticoagulant therapy. Studies focusing on patients with cancer were excluded, as were retrospective studies, meta-analyses, registries, and study designs that did not allow for the estimation of recurrence incidence, such as cross-sectional studies, case-control studies, and case reports.

### 2.3 Literature search

We conducted a systematic search of EMBASE, MEDLINE, and CENTRAL using predefined search terms. Detailed search strategies are provided in Supplementary Tables S1–S3. We also screened the reference lists of the included studies and consulted field experts to identify any additional relevant publications.

Duplicate records were removed prior to screening. In cases where multiple studies reported data from the same patient cohort, we included only the largest study that met our eligibility criteria.

Two independent reviewers (P.A.K. and L.E.) screened titles and abstracts for relevance. Discrepancies were resolved through discussion; if consensus could not be reached, a third reviewer (S.E.) provided the final decision. Full texts of eligible studies were then retrieved and evaluated by the same reviewers using the inclusion and exclusion criteria. All 3 reviewers are medical doctors with a strong research background in thrombosis.

Some observational cohort studies and controlled trials included both patients who had discontinued anticoagulation and those who remained anticoagulated throughout follow-up. For our analysis, we included only patients who had stopped anticoagulation.

In controlled trials comparing short- vs longer-term anticoagulation (eg, 6 weeks vs 6 months), only patients in the 6-month treatment group were included in the analysis.

### 2.4 Data extraction

Two reviewers (P.A.K. and L.E.) independently extracted data from the selected full-text articles using a standardized data collection form. Extracted information included the authors’ names, country, year of publication, study design, recruitment period, total number of participants, number of participants included in the analysis, participants’ characteristics (age and sex), location of the index VTE and classification as provoked or unprovoked (based on criteria defined in each study), and the type and duration of anticoagulation. Discrepancies were resolved through discussion with a third author (S.E.).

None of the published studies reported detailed data on patients with early recurrences or their characteristics. Therefore, we contacted the first or corresponding authors by email to request this information, including the number of early recurrences, patients’ age and sex, location of the index VTE, type and duration of initial anticoagulation, time from anticoagulation cessation to recurrence, and whether the recurrence occurred with or without a provoking factor.

A second follow-up email was sent if clarification was needed or if no response was received.

### 2.5 Risk of bias assessment

We assessed the incidence of early recurrent VTE in patients who had discontinued anticoagulation. Each study, or each relevant arm of a controlled trial, was treated as an independent observational cohort. To evaluate study quality, we used a modified version of the Newcastle-Ottawa Scale [14], which scores studies from 0 to 6 based on 3 selection and 3 outcome criteria: inclusion of a representative patient sample, confirmation that the outcome was absent at baseline, independent and blinded adjudication of outcomes, and adequacy of follow-up duration and completeness [1]. Risk of bias was assessed independently by 2 researchers (P.A.K. and S.E.). Studies scoring 5 or more points were considered to have a low risk of bias.

### 2.6 Outcome measures

The primary outcome was symptomatic recurrence of DVT of the lower limb and/or PE within 30 days of stopping anticoagulation. Recurrent DVT had to be diagnosed by compression ultrasound or venography, and recurrent PE had to be confirmed by spiral computed tomography or ventilation-perfusion scanning. We further evaluated potential risk factors for recurrence, including patient sex, age, location of the index event, and the presence or absence of a provoking factor.

### 2.7 Statistical analysis and data synthesis

For each study, we calculated the proportion of early recurrences and the corresponding 95% Clopper–Pearson CIs. A continuity correction of 0.5 was applied to studies with zero cell frequencies. We used the inverse-variance method to pool study data and estimate the overall proportion of early recurrences. To assess differences in recurrence rates, risk ratios (RRs) with 95% CIs were calculated and pooled using the Mantel–Haenszel method. Comparisons were made by sex, the presence or absence of a provoking factor, and the location of the index VTE. Since no common true effects can be assumed due to variation in study populations, random effects models were used to calculate the pooled rate and effects. Thus, we accounted not only for within-study (sampling) variance but also for between-study variation (heterogeneity). Estimation of the pooled RR with respect to patient age was not possible, as data were available only for patients with recurrences. Forest plots were generated to present the results of the meta-analyses. All statistical analyses were performed using R software (version 4.4.0, R Core Team; R Foundation for Statistical Computing, Vienna, Austria).

## 3 Results

**Figure 1:**
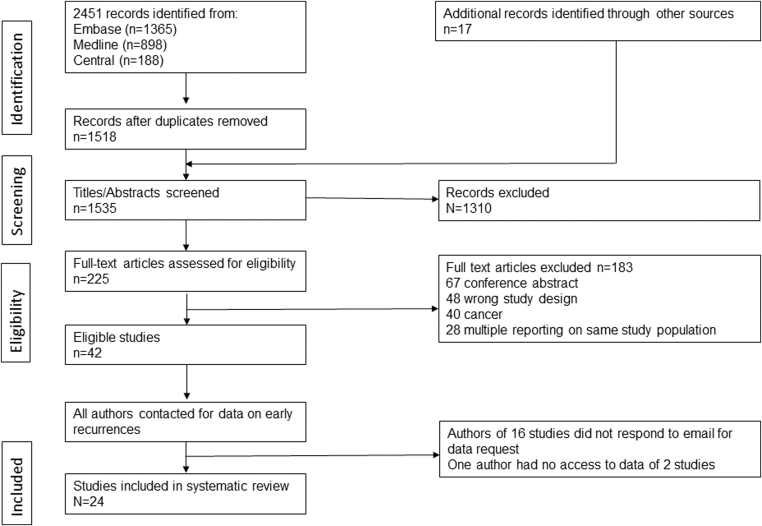
Search and selection of studies for systematic reviews according to the Preferred Reporting Items for Systematic Reviews and Meta-Analyses guidelines.

The study selection process is illustrated in Figure 1 in accordance with the Preferred Reporting Items for Systematic Reviews and Meta-Analyses guidelines. A total of 2451 records were identified through systematic database searches. After removing duplicates, 1535 unique records remained for title and abstract screening, based on predefined eligibility criteria. Following initial screening, 225 articles were selected for full-text assessment. Of these, 42 studies fulfilled the inclusion criteria and were considered for potential inclusion. However, none of the studies reported the incidence of early recurrent VTE and/or the characteristics of the affected patients; we therefore contacted the first or corresponding author of each of the 42 studies. Sixteen authors did not respond, and 2 datasets were no longer accessible. Ultimately, 24 studies provided sufficient data and were included in the final analysis of early recurrent VTE following cessation of anticoagulation.

### 3.1 Characteristics of studies and patient populations

Of the 24 studies included in the final analysis, 15 were prospective cohort studies [4,[15], [16], [17], [18], [19], [20], [21], [22], [23], [24], [25], [26], [27], [28]], and 9 were randomized controlled trials [[29], [30], [31], [32], [33], [34], [35], [36], [37]]. Detailed study characteristics are provided in Supplementary Table S4. Twenty-three studies reported on the risk of recurrent VTE after a first event [4,[15], [16], [17], [18], [19], [20], [21], [22], [23], [24], [25], [26], [27], [28], [29], [30], [31], [32], [33], [34], [35], [36]], and 1 study described the risk of recurrence after a second episode of VTE [37].

Twenty-three studies were conducted in Europe [4,[15], [16], [17], [18],[20], [21], [22], [23], [24], [25], [26], [27], [28], [29], [30], [31], [32], [33], [34], [35], [36], [37]] and 1 in Canada [19].

In total, 11,407 patients with either provoked or unprovoked VTE who had completed at least 3 months of initial anticoagulant therapy were included in the analysis. Detailed characteristics of the study populations are presented in the Table. Most patients received vitamin K antagonists as their initial anticoagulant treatment.

**Table:** Characteristics of study populations for assessing the risk of early recurrent venous thromboembolism.

Study	OAC stopped, *n*	Age (y), mean or median	Women (%)	Unprovoked VTE, *n* (%)	Location of VTE		Newcastle-Ottawa Scale Score (out of 6)
					DVT only, *n*	DVT + PE, *n*	
Boc et al. [15]	200	55	42	120 (60)	200	0	6
Bradbury et al. [29]	140	63	33	140 (100)	N.A.	N.A.	6
Cieslik et al. [16]	320	46	52	159 (50)	249	71	6
Couturaud et al. [30]	371	58	50	371 (100)	0	371	6
Couturaud et al. [31]	104	60	33	104 (100)	104	0	6
Di Minno et al. [17]	523	N.A.	N.A.	203 (38)	704	119	6
Eischer et al. [32]	32	N.A.	N.A.	32 (100)	19	13	6
Geersing et al. [33]	826	N.A.	N.A.	826 (100)	434	449	6
Jiménez et al. [18]	91	69	46	91 (100)	91	0	5
Kearon et al. [19]	392	N.A.	N.A.	N.A.	183	227	6
Kyrle et al. [20]	815	53	34	815 (100)	491	324	6
Kyrle et al. [4]	818	54	30	818 (100)	373	413	6
Palareti et al. [21]	599	67	50	282 (47)	484	115	6
Palareti et al. [34]	608	66	48 (100)	608 (100)	381	227	6
Palareti et al. [22]	1010	66	45	771	529	469	6
Palareti et al. [23]	612	N.A.	N.A.	N.A.	N.A.	N.A.	6
Poli et al. [24]	206	N.A.	N.A.	N.A.	0	239	6
Potaczek et al. [25]	229	45	49	229 (100)	115	114	6
Prandoni et al. [26]	1626	66	72	864 (53)	1073	553	6
Prandoni et al. [35]	538	64	50	306 (57)	N.A.	N.A.	6
Schulman et al. [36]	454	61	43	287 (63)	790	107	6
Schulman et al. [37]	111	65	37	89 (80)	193	34	6
Van Hylckama Vlieg et al. [27]	626	53	47	298 (48)	378	248	6
Zabczyk et al. [28]	156	44	47	89 (57)	0	156	6

DVT, deep vein thrombosis; N.A., not available; OAC, oral anticoagulation; PE, pulmonary embolism; VTE, venous thromboembolism.

### 3.2 Risk of bias

The individual components of the modified Newcastle-Ottawa Scale for all the included studies are summarized in Supplementary Table S5. Overall, the risk of bias was considered low, as no study received a score below 5 (Table).

### 3.3 Incidence and risk factors of early recurrent VTE

**Figure 2:**
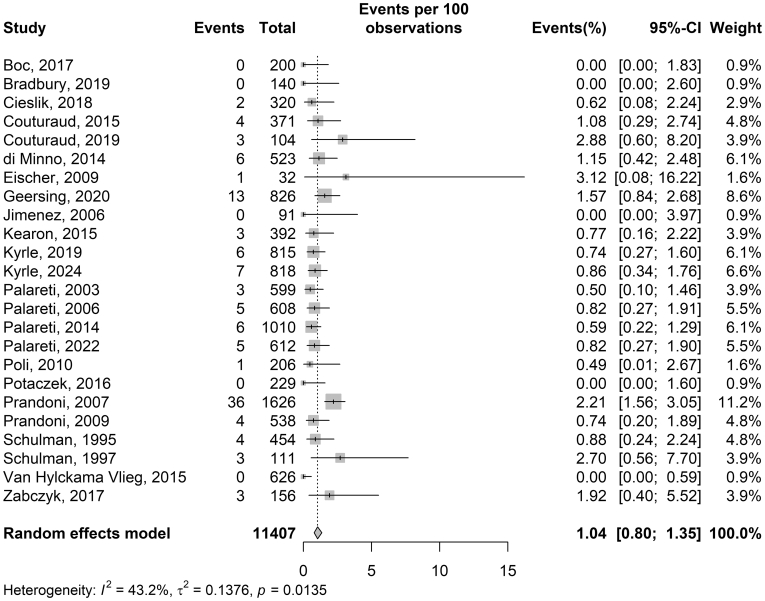
Rates of early recurrent venous thromboembolism after stopping anticoagulation.

Among the 11,407 patients included in the analysis, 115 (1.01%) experienced an early recurrence of lower extremity DVT or PE. The pooled incidence of early recurrence was 1.04% (95% CI, 0.8%-1.4%; I^2^ = 43%; Figure 2). Notably, 5 of the 24 included studies reported no early recurrent events. Of the 115 recurrent events, 51 were PE, and importantly, no fatal events were reported. Of 64 patients with an index DVT, 52 (81%) recurred as DVT; of 51 patients with an index PE, 38 (75%) recurred as PE.

Patients with an unprovoked index VTE had a significantly higher risk of early recurrence than those with a provoked event (pooled RR, 2.6; 95% CI, 1.4-4.6; *P* = .001; I^2^ = 0%). The pooled incidence of early recurrence among patients with an unprovoked VTE was 1.32% (95% CI, 0.95%-1.8%; I^2^ = 54%; Supplementary Figure).

The risk of early recurrent VTE was not significantly higher in men than in women, with a pooled RR of 1.2 (95% CI, 0.6-2.3; *P* = .7; I^2^ = 40%). Patients with DVT at presentation had a similar risk of recurrence to those with incident PE (pooled RR, 0.6; 95% CI, 0.3-1.2; *P* = .1; I^2^ = 15%).

Findings regarding advanced age as a risk factor were inconsistent. Among 13 studies reporting the age distribution of both the overall cohort discontinuing oral anticoagulation and the subset with early recurrences, 5 found younger ages in the early recurrence group, 6 found older ages, and 2 reported no age difference.

## 4 Discussion

Determining the optimal duration of anticoagulation is a critical aspect of managing patients with a history of VTE. Strategies to identify patients at high risk of recurrence who may benefit from extended anticoagulation, and those at low risk in whom the bleeding risk may outweigh the benefits of anticoagulation, often involve measuring markers of coagulation activation, primarily D-dimer. These measurements are performed either during anticoagulant therapy or, more commonly, after its discontinuation once patients have completed at least 3 months of treatment. However, interrupting anticoagulation, even briefly, increases the risk of early recurrent VTE and may be particularly harmful to patients at high risk of recurrence.

In this systematic review, we found that the overall risk of recurrent VTE within the first 30 days after anticoagulation cessation is low. Among 11,407 patients with VTE, only 115 experienced early recurrence of DVT or PE, yielding a pooled recurrence rate of 1.04%. Importantly, none of these recurrent events was fatal.

Our analysis also revealed that patients with VTE were provoked by a transient risk factor and had a lower risk of early recurrence compared with those with unprovoked VTE. Men had a higher risk of recurrence than women. The effect of advanced age on the risk of early recurrence remains unclear. These findings of short-term recurrence mirror established data on long-term risk, in which unprovoked events and male sex are recognized as key predictors, whereas the role of age remains uncertain.

The risk of recurrence among patients with VTE varies considerably between individuals, making the optimal duration of anticoagulation uncertain. While risk stratification tools have proven useful in guiding the duration of therapy, concerns about early recurrence following interruption of anticoagulation may limit the practical implementation of this strategy. One risk assessment tool, the Vienna Prediction Model [3], has recently been introduced and shows promise in identifying low-risk patients who may not require extended anticoagulation. This model has been validated in 2 large management studies [4,33] and is now considered suitable for clinical use. However, it requires temporary interruption of anticoagulation shortly after treatment cessation, which inherently carries a risk of early recurrence.

The low incidence of early recurrent VTE, occurring in only 1.32% of patients with an unprovoked index VTE, with no fatal events, is reassuring and supports the careful use of this approach. This is especially relevant as current guidelines favor extended anticoagulation for all patients with unprovoked VTE, thereby exposing many to prolonged bleeding risk as well as lifestyle limitations and psychological burdens. An individualized strategy involving temporary cessation of anticoagulation for risk assessment could potentially reduce unnecessary treatment and its associated risks in a substantial number of patients.

### 4.1 Strengths

This study has several notable strengths. We adhered to a preregistered protocol (PROSPERO) and applied rigorous, predefined search criteria to identify all eligible studies, thereby minimizing selection bias. To ensure completeness, we contacted all first or corresponding authors for additional data, as none of the included studies reported the incidence of early recurrent VTE or detailed patient characteristics. We excluded retrospective studies, meta-analyses, registries, and other designs unsuitable for estimating recurrent VTE incidence (eg, cross-sectional studies, case-control studies, and case reports). Only studies involving noncancer populations with objectively verified endpoints were included. The risk of bias was assessed using the modified Newcastle-Ottawa Scale and was considered negligible overall.

### 4.2 Limitations

The main limitation was the moderate response rate, as data were obtained for only 24 of 42 eligible studies. This likely reflects the extended 30-year search period and the difficulty of contacting some of the original investigators. However, this 57% inclusion rate may have introduced selection bias if studies with particularly high or low recurrence rates were less likely to provide data. The long timeframe also introduces potential variability in diagnostic approaches, anticoagulant use, and definitions of provoked vs unprovoked VTE. Age-specific pooled risk estimates could not be calculated due to incomplete data. Similarly, other potentially relevant predictors, including body mass index, thrombophilia, and family history, could not be assessed because they were often unreported. Data on 30-day all-cause mortality were unavailable, which may represent a competing event with recurrent VTE and could have influenced the observed recurrence rates. Patients with upper extremity DVT were not included; therefore, the findings cannot be generalized to this population. Most included studies were conducted in Europe, with only 1 from North America, which may limit the applicability of our findings to settings with different ethnicities, healthcare systems, and VTE management practices.

## 5 Conclusion

The risk of recurrent DVT or PE within 30 days of anticoagulation cessation is low. Strategies that estimate the optimal duration of anticoagulation by temporarily interrupting therapy appear to be safe. Nonetheless, it is essential to engage patients in a thorough discussion that clearly communicates both the potential risks and benefits of this approach.
