# Supplementary material for: The incidence of early recurrent venous thromboembolism: a systematic review and meta-analysis
Source: Res Pract Thromb Haemost. 2025 Dec 29;10(1):103317. doi: 10.1016/j.rpth.2025.103317 (PMC12860354; doi:10.1016/j.rpth.2025.103317)

**SUPPLEMENTARY MATERIAL**

**Eischer L, et al.**

**The incidence of early recurrent venous thromboembolism:
 a systematic review and meta-analysis**

**Supplementary Table S1:** Literature search strategy for EMBASE

|  | Search processing: 19-04-2022 |  |
| --- | --- | --- |
| #25 | #24 NOT cancer*:ti | **1,365** |
| #24 | #23 NOT 'case report'/exp | **1,530** |
| #23 | #19 NOT #22 | **1,601** |
| #22 | #20 NOT #21 | **3,124,387** |
| #21 | 'adult'/exp OR adult*:ti,ab,kw,de | **10,792,634** |
| #20 | 'juvenile'/exp OR child*:ti,ab,kw,de OR infan*:ti,ab,kw,de OR juvenile*:ti,ab,kw,de OR adolescen*:ti,ab,kw,de OR pediatric*:ti,ab,kw,de OR paediatric*:ti,ab,kw,de | **5,052,610** |
| #19 | #10 AND #18 | **1,640** |
| #18 | #11 OR #12 OR #13 OR #14 OR #15 OR #16 OR #17 | **1,495,897** |
| #17 | ('risk assessment'/exp OR ((risk NEAR/5 assess*):ti,ab,kw,de)) AND (predict*:ti,ab,kw,de OR prognos*:ti,ab,kw,de OR prevent*:ti,ab,kw,de OR prophyla*:ti,ab,kw,de OR prospectiv*:ti,ab,kw,de) | **338,327** |
| #16 | tailor*:ti,ab,kw | **145,200** |
| #15 | ((predict* OR prognos*) NEAR/5 (recurren* OR recidiv* OR factor* OR model* OR test* OR risk* OR tool* OR score*)):ti,ab,kw,de | **890,064** |
| #14 | (secondary* NEAR/2 (prevent* OR prophyla*)):ti,ab,kw,de | **58,770** |
| #13 | (predict*:ti,ab,kw,de OR prognos*:ti,ab,kw,de OR prospectiv*:ti,ab,kw,de) AND (recurren*:ti,ab,kw,de OR recidiv*:ti,ab,kw,de) AND risk*:ti,ab,kw,de | **148,229** |
| #12 | ((risk* OR prevent* OR prophyla*) NEAR/3 (recurren* OR recidiv*)):ti,ab,kw | **105,798** |
| #11 | 'recurrence risk'/exp | **86,456** |
| #10 | #4 AND #9 | **3,635** |
| #9 | #5 OR #6 OR #7 OR #8 | **9,620** |
| #8 | ((unprovoked OR idiopathic OR initial* OR first OR spontaneous*) NEAR/5 (lung OR pulmonary) NEAR/3 (emboli* OR microemboli*)):ti,ab,kw | **1,615** |
| #7 | ((unprovoked OR idiopathic OR initial* OR first OR spontaneous*) NEAR/5 (venous OR vein OR veins OR vena OR lung OR pulmonary) NEAR/3 (thromboemboli* OR 'thrombo emboli*' OR thrombosis* OR thrombophlebit* OR 'thrombo phlebit*' OR thrombus)):ti,ab,kw | **4,932** |
| #6 | ((unprovoked OR idiopathic OR initial* OR first OR spontaneous*) NEAR/5 (vte OR dvt)):ti,ab,kw | **4,611** |
| #5 | 'unprovoked venous thromboembolism'/exp | **11** |
| #4 | #1 OR #2 OR #3 | **161,847** |
| #3 | anticoagulation:ti,ab,kw OR 'anti coagulation':ti,ab,kw | **85,461** |
| #2 | ((anticoagula* OR 'anti coagula*') NEAR/3 (therapy* OR treatment*)):ti,ab,kw | **45,738** |
| #1 | 'anticoagulant therapy'/exp OR 'anticoagulation'/exp | **124,835** |

**Supplementary Table S2:** Literature search strategy for MEDLINE

|  | Search processing: 19-04-2022 |  |
| --- | --- | --- |
| 1 | exp Anticoagulants/ | 235640 |
| 2 | ((anticoagula* or 'anti coagula*') adj3 (therapy* or treatment*)).ti,ab,kf. | 29064 |
| 3 | (anticoagulation or 'anti coagulation').ti,ab,kf. | 48775 |
| 4 | 1 or 2 or 3 | 263169 |
| 5 | ((unprovoked or idiopathic or initial* or first or spontaneous*) adj5 (vte or dvt)).ti,ab,kf. | 2230 |
| 6 | ((unprovoked or idiopathic or initial* or first or spontaneous*) adj5 (venous or vein or veins or vena or lung or pulmonary) adj3 (thromboemboli* or 'thrombo emboli*' or thrombosis* or thrombophlebit* or 'thrombo phlebit*' or thrombus)).ti,ab,kf. | 3079 |
| 7 | ((unprovoked or idiopathic or initial* or first or spontaneous*) adj5 (lung or pulmonary) adj3 (emboli* or microemboli*)).ti,ab,kf. | 906 |
| 8 | 5 or 6 or 7 | 5414 |
| 9 | 4 and 8 | 2446 |
| 10 | ((risk* or prevent* or prophyla*) adj3 (recurren* or recidiv*)).ti,ab,kf. | 68337 |
| 11 | ((predict* or prognos* or prospectiv*) and (recurren* or recidiv*) and risk*).ti,ab,kf,hw. | 80974 |
| 12 | (secondary* adj2 (prevent* or prophyla*)).ti,ab,kf,hw. | 46789 |
| 13 | ((predict* or prognos*) adj5 (recurren* or recidiv* or factor* or model* or test* or risk* or tool* or score*)).ti,ab,kf,hw. | 831090 |
| 14 | tailor*.ti,ab,kf. | 115283 |
| 15 | (exp Risk Assessment/ or (risk adj5 assess*).ti,ab,kf,hw.) and (predict* or prognos* or prevent* or prophyla* or prospectiv*).ti,ab,kf,hw. | 192873 |
| 16 | 10 or 11 or 12 or 13 or 14 or 15 | 1167425 |
| 17 | 9 and 16 | 1033 |
| 18 | exp adolescent/ or exp child/ or (child* or infan* or juvenile* or adolescen* or pediatric* or paediatric*).ti,ab,kf,hw. | 4470699 |
| 19 | exp Adult/ or adult*.ti,ab,kf,hw. | 8474464 |
| 20 | 18 not 19 | 2400912 |
| 21 | 17 not 20 | 1015 |
| 22 | 21 not exp case reports/ | 982 |
| 23 | 22 not cancer*.ti. | 898 |

**Supplementary Table S3:** Literature search strategy for CENTRAL

|  | Search processing: 19-04-2022 |  |
| --- | --- | --- |
| 1 | exp Anticoagulants/ | 12006 |
| 2 | ((anticoagula* or 'anti coagula*') adj3 (therapy* or treatment*)).ti,ab,kw. | 4835 |
| 3 | (anticoagulation or 'anti coagulation').ti,ab,kw. | 7125 |
| 4 | 1 or 2 or 3 | 18998 |
| 5 | ((unprovoked or idiopathic or initial* or first or spontaneous*) adj5 (vte or dvt)).ti,ab,kw. | 422 |
| 6 | ((unprovoked or idiopathic or initial* or first or spontaneous*) adj5 (venous or vein or veins or vena or lung or pulmonary) adj3 (thromboemboli* or 'thrombo emboli*' or thrombosis* or thrombophlebit* or 'thrombo phlebit*' or thrombus)).ti,ab,kw. | 403 |
| 7 | ((unprovoked or idiopathic or initial* or first or spontaneous*) adj5 (lung or pulmonary) adj3 (emboli* or microemboli*)).ti,ab,kw. | 90 |
| 8 | 5 or 6 or 7 | 744 |
| 9 | 4 and 8 | 402 |
| 10 | ((risk* or prevent* or prophyla*) adj3 (recurren* or recidiv*)).ti,ab,kw. | 16133 |
| 11 | ((predict* or prognos* or prospectiv*) and (recurren* or recidiv*) and risk*).ti,ab,kw,hw. | 11566 |
| 12 | (secondary* adj2 (prevent* or prophyla*)).ti,ab,kw,hw. | 8727 |
| 13 | ((predict* or prognos*) adj5 (recurren* or recidiv* or factor* or model* or test* or risk* or tool* or score*)).ti,ab,kw,hw. | 47151 |
| 14 | tailor*.ti,ab,kw. | 15220 |
| 15 | (exp Risk Assessment/ or (risk adj5 assess*).ti,ab,kw,hw.) and (predict* or prognos* or prevent* or prophyla* or prospectiv*).ti,ab,kw,hw. | 20891 |
| 16 | 10 or 11 or 12 or 13 or 14 or 15 | 100610 |
| 17 | 9 and 16 | 210 |
| 18 | 17 not cancer*.ti. | 188 |

**Supplementary Table S4**: Characteristics of included studies

| Study | County | Study design | Recruitment  Period, year | Total study population,  N | Age, years mean or median | Women,  (%) | Unprovoked VTE,  N (%) | Location of VTE  DVT only, N PE, N | | OAC stopped,  N | Newcastle-Ottawa scale score (out of 6) |
| --- | --- | --- | --- | --- | --- | --- | --- | --- | --- | --- | --- |
|  |  |  |  |  |  |  |  |  |  |  |  |
| Boc 2017 | Slovenia | Prospective cohort study | 2008-2010 | 200 | 55 | 42 | 120 (60) | 200 | 0 | 200 | 6 |
| Bradbury 2019 | Great Britain | Controlled trial | 2011-2015 | 273 | 63 | 33 | 140 (100) | n.a. | n.a. | 140 | 6 |
| Cieslik 2018 | Poland | Prospective cohort study | 2009-2010 | 320 | 46 | 52 | 159 (50) | 249 | 71 | 320 | 6 |
| Couturaud 2015 | France | Controlled trial | 2007-2012 | 371 | 58 | 50 | 371 (100) | 0 | 371 | 371 | 6 |
| Couturaud 2019 | France | Controlled trial | 2007-2013 | 104 | 60 | 33 | 104 (100) | 104 | 0 | 104 | 6 |
| Di Minno 2014 | Italy | Prospective cohort study | 1993-2003 | 823 | 48 | 58 | 321(39) | 704 | 119 | 523 | 6 |
| Eischer 2009 | Austria | Controlled trial | 2001-2004 | 34 | 54 | 68 | 34 (100) | 20 | 14 | 32 | 6 |
| Geersing 2020 | Netherlands | Controlled trial | 2011-2015 | 883 | 55 | 43 | 883 (100) | 434 | 449 | 826 | 6 |
| Jimenez 2006 | Spain | Prospective cohort study | 2000-2002 | 91 | 69 | 46 | 91 (10) | 91 | 0 | 91 | 5 |
| Kearon 2015 | Canada | Prospective management study | 2008-2013 | 410 | 51 | 46 | 340 (83) | 183 | 227 | 392 | 6 |
| Kyrle 2019 | Austria | Prospective cohort study | 1992-2009 | 815 | 53 | 34 | 815 (100) | 491 | 324 | 815 | 6 |
| Kyrle 2024 | Austria | Prospective cohort study | 2013-2019 | 818 | 54 | 30 | 818 (100) | 373* | 413* | 818 | 6 |
| Palareti 2003 | Italy | Prospective cohort study | 1995-2002 | 599 | 67 | 50 | 282 (47) | 484 | 115 | 599 | 6 |
| Palareti 2006 | Italy | Controlled trial | 2002-2005 | 608 | 66 | 48 | 608 (100) | 381 | 227 | 608 | 6 |
| Palareti 2014 | Italy | Prospective cohort study | 2008-2011 | 1010 | 66 | 45 | 771 (7) | 529* | 469* | 1010 | 6 |
| Palareti 2022 | Italy | Prospective Management study | n.a. | 732 | 59 | 37 | 555 (76) | n.a. | n.a. | 612 | 6 |
| Poli 2010 | Italy | Prospective cohort study | n.a. | 239 | 59 | 51 | 161 (67) | 0 | 239 | 206 | 6 |
| Potaczek 2016 | Poland | Prospective cohort study | 2010-2012 | 229 | 45 | 49 | 229 (100) | 115 | 114 | 229 | 6 |
| Prandoni 2007 | Italy | Prospective cohort study | 1991-2003 | 1626 | 66 | 72 | 864 (53) | 1073 | 553 | 1626 | 6 |
| Prandoni 2009 | Italy | Controlled trial | 1999-2003 | 538 | 64 | 50 | 306 (57) | n.a. | n.a. | 538 | 6 |
| Schulman 1995 | Sweden | Controlled trial | 1988-1991 | 902 | 61 | 44 | 553 (61) | 790 | 107 | 454 | 6 |
| Schulman 1997 | Sweden | Controlled trial | 1988-1991 | 227 | 64 | 39 | 184 (81) | 193 | 34 | 111 | 6 |
| Van Hylckama Vlieg 2015 | Netherlands | Prospective cohort study | 2003-2008 | 626 | 53 | 47 | 298 (48) | 378 | 248 | 626 | 6 |
| Zabczyk 2017 | Poland | Prospective cohort study | 2009-2012 | 156 | 44 | 47 | 89 (57) | 0 | 156 | 156 | 6 |

* does not add up to total number of patients due to missing data

OAC: oral anticoagulation, VTE: venous thromboembolism, DVT: deep vein thrombosis, PE: pulmonary embolism, n.a. not available

**Supplementary Table S5:** Risk of bias assessment using the modified Newcastle-Ottawa scale

| Study | Selection | | | Outcome | | |  |
| --- | --- | --- | --- | --- | --- | --- | --- |
|  |  |  |  |  |  |  |  |
|  | Consecutive patients with objectively diagnosed VTE | Patients have completed at least 3 months of initial anticoagulation | Demonstration that no patient had recurrent VTE at start of follow-up | Primary outcome assessment of recurrent VTE | Acceptable length of follow-up after stopping anticoagulation | Follow-up was sufficiently complete after stopping anticoagulation | Modified Newcastle Ottawa scale Overall score (out of 6) |
|  |  |  |  |  |  |  |  |
| Boc, 2017 | + | + | + | + | + | + | 6 |
| Bradbury 2019 | + | + | + | + | + | + | 6 |
| Cieslik, 2018 | + | + | + | + | + | + | 6 |
| Couturaud, 2015 | + | + | + | + | + | + | 6 |
| Couturaud, 2019 | + | + | + | + | + | + | 6 |
| Di Minno 2014 | + | + | + | + | + | + | 6 |
| Geersing, 2020 | + | + | + | + | + | + | 6 |
| Jimenez, 2006 | + | - | + | + | + | + | 5 |
| Kearon, 2015 | + | + | + | + | + | + | 6 |
| Kyrle, 2019 | + | + | + | + | + | + | 6 |
| Kyrle, 2024 | + | + | + | + | + | + | 6 |
| Eischer, 2009 | + | + | + | + | + | + | 6 |
| Palareti, 2002 | + | + | + | + | + | + | 6 |
| Palareti, 2006 | + | + | + | + | + | + | 6 |
| Palareti, 2014 | + | + | + | + | + | + | 6 |
| Palareti, 2022 | + | + | + | + | + | + | 6 |
| Poli, 2010 | + | + | + | + | + | + | 6 |
| Potaczek, 2016 | + | + | + | + | + | + | 6 |
| Prandoni, 2007 | + | + | + | + | + | + | 6 |
| Prandoni, 2009 | + | + | + | + | + | + | 6 |
| Schulman, 1995 | + | + | + | + | + | + | 6 |
| Schulman, 1997 | + | + | + | + | + | + | 6 |
| V. Hylckama Vlieg, 2015 | + | + | + | + | + | + | 6 |
| Zabczyk, 2017 | + | + | + | + | + | + | 6 |

VTE: venous thromboembolism

**Supplementary Figure:** Rates of early recurrent venous thromboembolism in patients with an unprovoked index event after stopping anticoagulation.

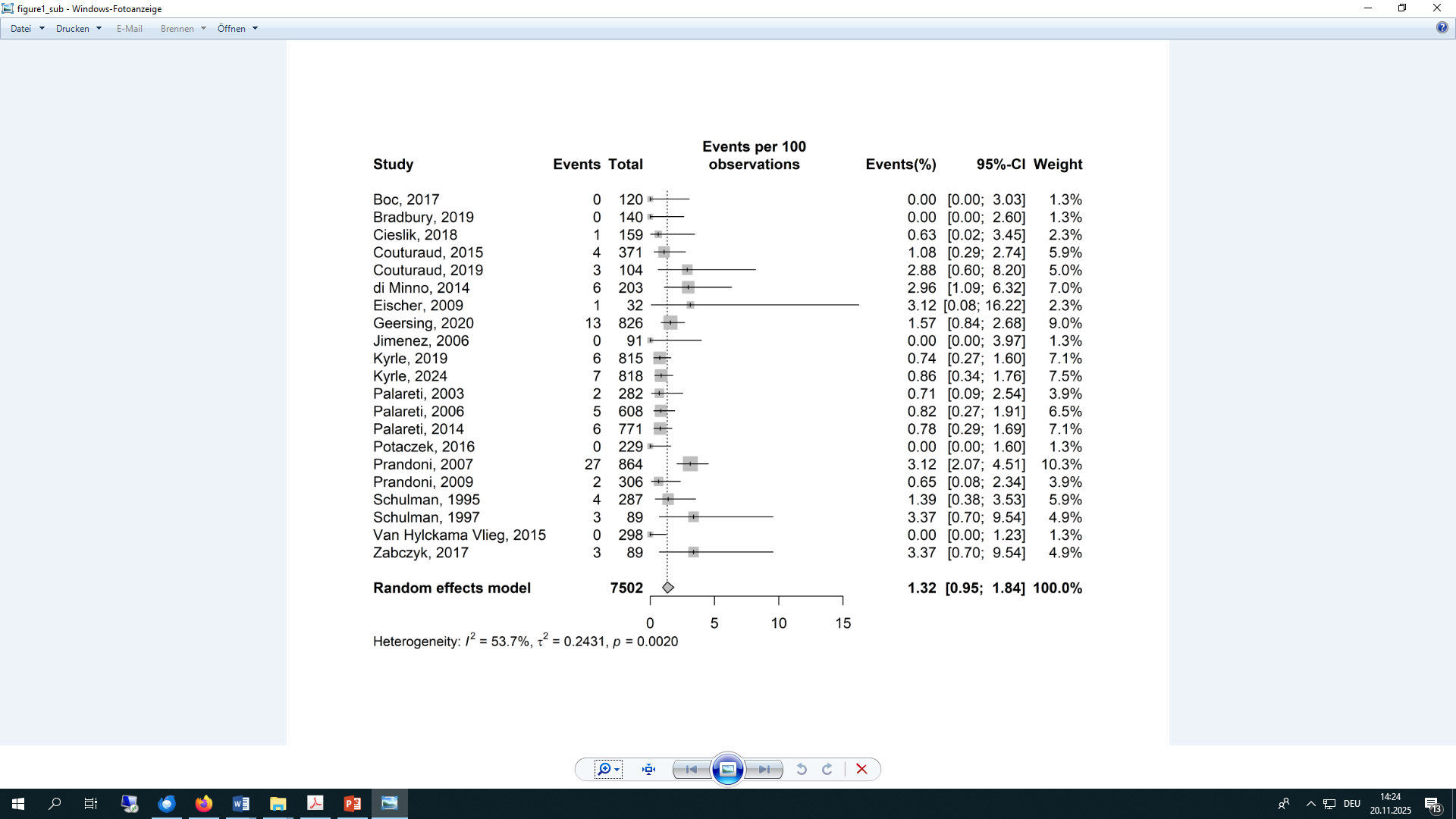

Supplement: Supplementary Material [file mmc1.docx]
